# Obtaining Thickness Maps of Corneal Layers Using the Optimal Algorithm for Intracorneal Layer Segmentation

**DOI:** 10.1155/2016/1420230

**Published:** 2016-05-09

**Authors:** Hossein Rabbani, Rahele Kafieh, Mahdi Kazemian Jahromi, Sahar Jorjandi, Alireza Mehri Dehnavi, Fedra Hajizadeh, Alireza Peyman

**Affiliations:** ^1^Department of Bioelectrics and Biomedical Engineering, School of Advanced Technologies in Medicine, Medical Image and Signal Processing Research Center, Isfahan University of Medical Sciences, Isfahan 8174673461, Iran; ^2^Student Research Center, School of Advanced Technologies in Medicine, Isfahan University of Medical Sciences, Isfahan 8174673461, Iran; ^3^Noor Ophthalmology Research Center, Noor Eye Hospital, Tehran 1968653111, Iran; ^4^Isfahan University of Medical Sciences, Isfahan 817467346, Iran

## Abstract

Optical Coherence Tomography (OCT) is one of the most informative methodologies in ophthalmology and provides cross sectional images from anterior and posterior segments of the eye. Corneal diseases can be diagnosed by these images and corneal thickness maps can also assist in the treatment and diagnosis. The need for automatic segmentation of cross sectional images is inevitable since manual segmentation is time consuming and imprecise. In this paper, segmentation methods such as Gaussian Mixture Model (GMM), Graph Cut, and Level Set are used for automatic segmentation of three clinically important corneal layer boundaries on OCT images. Using the segmentation of the boundaries in three-dimensional corneal data, we obtained thickness maps of the layers which are created by these borders. Mean and standard deviation of the thickness values for normal subjects in epithelial, stromal, and whole cornea are calculated in central, superior, inferior, nasal, and temporal zones (centered on the center of pupil). To evaluate our approach, the automatic boundary results are compared with the boundaries segmented manually by two corneal specialists. The quantitative results show that GMM method segments the desired boundaries with the best accuracy.

## 1. Introduction

Optical Coherence Tomography (OCT) is one of the most informative methodologies in ophthalmology today. It works noninvasively, has no contact, and provides cross sectional images from anterior and posterior segments of the eye. Imaging of the anterior segment is needed in refractive surgery and contact lens implantation [1].

Cornea was first imaged by OCT in 1994 [2] with similar wavelength of the light as retina (830 nm). A longer wavelength of 1310 nm with advantage of better penetration through sclera as well as real-time imaging at 8 frames per second was proposed in 2001 [3]. Specific systems for visualization of anterior eye (anterior segment OCT, ASOCT) were commercially available in 2011 [4] and there are three main producers for this device: SL-OCT (Heidelberg Engineering), the Visante (Carl Zeiss Meditec, Inc.), and CASIA (Tomey, Tokyo, Japan). Furthermore, many devices based on Fourier domain OCT (FDOCT) obtain images from both anterior and posterior segments using the shorter wavelength of 830–870 nm. The main competitor device to OCT in AS imaging is ultrasound biomicroscopy (UBM). The UBM method can visualize some anatomical structures posterior to iris; on the other hand, good quality of the images in this method is really dependant on the operator's skill [5].

The adult cornea is approximately 0.5 millimeter thick at the center and it gradually increases in thickness toward the periphery. The human cornea is comprised of five layers: Epithelium, Bowman's membrane, stroma, Descemet's membrane, and the Endothelium [5].

Some corneal diseases need to be diagnosed by precise evaluation of subcorneal layers. A good example for need of thickness mapping in subcorneal layers is diseases like Keratoconus. In this illness, the thickness of the Epithelium becomes altered to reduce corneal surface irregularity [6]. As a result, the presence of an irregular stroma cannot be diagnosed by observing the thickness map of the whole cornea. Therefore, analysis of epithelial and stromal thicknesses and shapes separately can improve the diagnosis [7, 8].

Several methods, like confocal microscopy, ultrasound, and OCT, have already been used to measure the corneal epithelial thickness. The average central Epithelium thickness is used in many studies [9–12]. Very high-frequency ultrasound is used to map the corneal Epithelium and stromal thickness [7]. The mentioned two methods had their drawbacks; namely, confocal microscopy is an invasive method and ultrasound method needs immersion of the eye in a coupling fluid [7, 13–16].

Accurate segmentation of corneal boundaries is necessary for production of correct thickness maps. An error of several micrometers can lead to wrong diagnosis. The large volume of these data in clinical evaluation makes manual segmentation time consuming and impractical [17–19].

Current methods for segmentation of cornea can be summarized as below.

Graglia et al. [20] proposed an approach for contour detection algorithm for finding Epithelium and Endothelium points and tracing the contour of the cornea pixel by pixel from these two points with a weight criterion. Eichel et al. [21] proposed a semiautomated segmentation algorithm for extraction of five corneal boundaries using a global optimization method. Li et al. [22–24] proposed an automatic method for corneal segmentation using a combination of fast active contour (FAC) and second-order polynomial fitting algorithm. Eichel et al. [18, 25] proposed a semiautomatic method for corneal segmentation by utilizing Enhanced Intelligent Scissors (EIS) and user interaction. Shen et al. [26] used a threshold-based technique which failed in segmentation of posterior surface of the cornea. In Williams et al.'s study [27], Level Set segmentation is investigated to obtain good results with low speed. LaRocca et al. [19] proposed an automatic algorithm to segment boundaries of three corneal layers using graph theory and dynamic programming. This method segments three clinically important corneal layer boundaries (Epithelium, Bowman, and Endothelium). Their results had good agreement with manual observers only for the central region of the cornea where the highest signal to noise ratio was found. A 3D approach is also proposed by Robles et al. [28] to segment three main corneal boundaries by graph-based method. In a more recent work by Williams et al. [29], a Graph Cut based segmentation technique is proposed to improve the speed and efficiency of the segmentation. Previous methods were never a perfect method for segmentation. Some of them suffered from low number of corneal images for testing, and others lacked precision in low contrast to noise ratios. Furthermore, none of previous works demonstrated a thickness map of corneal region for sublayers.

In this paper, we segment the boundaries of corneal layers by utilizing Graph Cut (GC), Gaussian Mixture Model (GMM), and Level Set (LS) methods. We evaluate the performance of segmenting Epithelium, Bowman, and Endothelium boundaries in OCT images using these segmentation methods. Finally, using the extracted boundaries of 3D corneal data, the 3D thickness maps of each layer are obtained.

**Figure 1:**
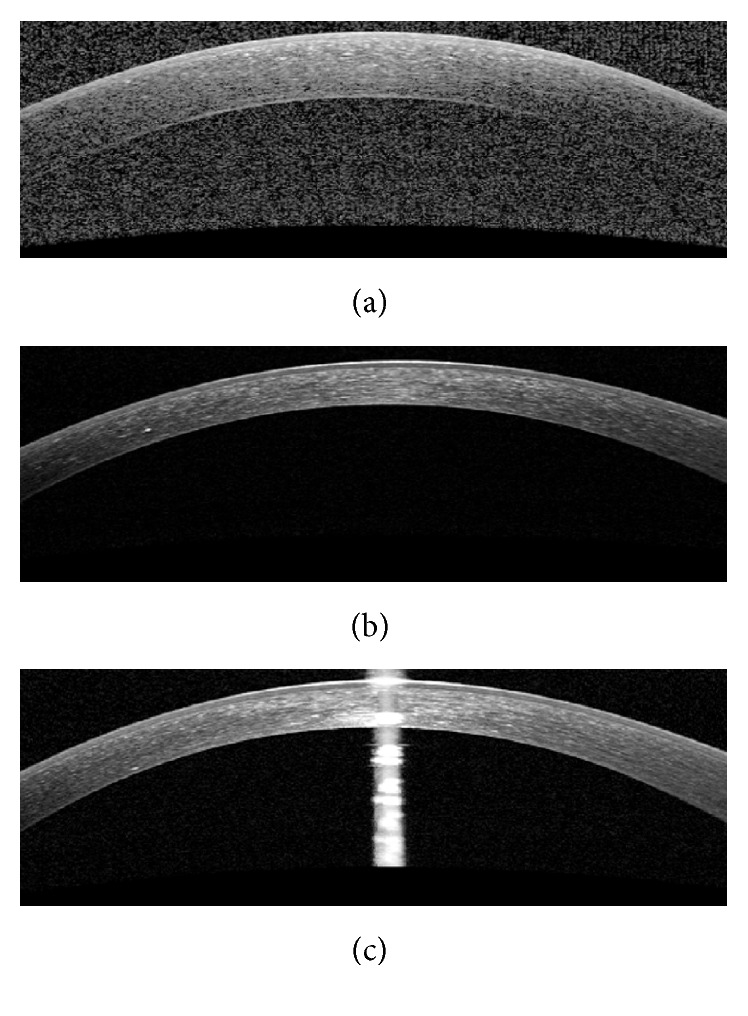
Examples of corneal images of varying signal to noise ratio (SNR) used in this study. (a) High SNR corneal image. (b) Low SNR corneal image. (c) Corneal image with central artifact.

The OCT images captured from high-tech devices may have high SNR (Figure 1(a)), but in many cases, they have a low SNR (Figure 1(b)). Furthermore, some of OCT images may be affected by different types of artifact like central artifact (Figure 1(c)). The central artifact is the vertical saturation artifact that occurs around the center of the cornea due to the back-reflections from the corneal apex, which saturates the spectrometer line camera [19].

## 2. Theory of Algorithms

In this section we explain the theory of GMM, GC, and LS algorithms.

### 2.1. GMM

As we explained in [30], GMM can be used for modeling of cornea layers in OCT images. For *D*-dimensional Gaussian random vector $\vec{x}$ with mean vector ${\vec{\mu }}_{i}$ and covariance matrix ${\vec{\Sigma }}_{i}$ given below

(1) bix→=12πD/2Σi1/2exp⁡−12x→−μ→i′Σi−1x→−μ→i

a weighted mixture of *M* Gaussian distribution would be

(2) $$\begin{array}{c} p\left(\;\vec{x}\;|\;\lambda \;\right)=\overset{M}{\underset{i=1}{\sum }}p_{i}b_{i}\left(\;\vec{x}\;\right), \end{array}$$

where *p*_*i*_s are the weights of mixture components and

(3) $$\begin{array}{c} \overset{M}{\underset{i=1}{\sum }}p_{i}=1. \end{array}$$

Parameters of GMM *λ* = {*μ*_*i*_, *p*_*i*_, Σ_*i*_} are calculated using iterative expectation maximization (EM) algorithm as follows (for *T* training vectors):

(4) pi ∣ x→t,λ=pibix→t∑k=1Mpkbkx→t,

(5) p−i=1T∑t=1Tpi ∣ x→t,λ,

(6) μ−→i=∑t=1Tpi ∣ x→t,λx→t∑t=1Tpi ∣ x→t,λ,

(7) σ−i2=∑t=1Tpi ∣ x→t,λx→t2∑t=1Tpi ∣ x→t,λ−μ→i2.

### 2.2. GC

We use normalized cuts for segmentation which is explained by Shi and Malik [31]. This criterion measures both the total dissimilarity between the different groups and the total similarity within the groups. Suppose we have a graph *G* = (*V*, *E*) which is composed of two distinct parts of *A*, *B* and easily achieved by removing edges between these two sectors and has the following property: *A*, *B*, *A* ∪ *B* = *V*, *A*∩*B* = *ϕ*. Based on the total weight of the edges that are removed, we can calculate the degree of dissimilarity between these two parts which is called cut:

(8) $$\begin{array}{c} cut\left(\;A,B\;\right)=\underset{u\in A,v\in B}{\sum }w\left(\;u,v\;\right), \end{array}$$

where *w*(*u*, *v*) is the edge weight between nodes *u* and *v*. Here the graph is divided into two subsections in such a way that the total weight of edges connecting these two parts is smaller than any other division. Instead of looking at the value of total edge weight connecting the two partitions, the cut cost as a fraction of the total edge connections will be computed to all the nodes in the graph which is called normalized cut (Ncut):

(9) $$\begin{array}{c} Ncut\left(\;A,B\;\right)=\frac{cut\left(A,B\right)}{assoc\left(A,V\right)}+\frac{cut\left(A,B\right)}{assoc\left(B,V\right)}, \end{array}$$

where assoc(*A*, *V*) = ∑_*u*∈*A*,*t*∈*V*_ *w*(*u*, *t*) is the total connection from nodes in *A* to all nodes in the graph and assoc(*B*, *V*) is similarly defined. Optimal cut is the cut which minimizes these criteria. In other words, minimizing the dissimilarity of the two subsections is equal to the maximizing of similarity within each subsection.

Suppose there is an optimal decomposition of a graph with vertices *V* to the components *A* and *B* based on the criteria of the optimal cut. Consider the following generalized eigenvalue problem:

(10) $$\begin{array}{c} \left(\;D-W\;\right)y=\lambda Dy, \end{array}$$

where *D* is an *N* × *N* diagonal matrix with *d* on its diagonal, *d*(*i*) = ∑_*j*_ *w*(*i*, *j*) is the total connection from node *i* to all other nodes, and *W* is an *N* × *N* symmetrical matrix which contains the edge weights. We solve this equation for eigenvectors with the smallest eigenvalues. Then, the eigenvector with the second smallest eigenvalue is used to bipartition the graph. The divided parts, if necessary, will be divided again.

This algorithm has also been used in the processing of fundus images [32, 33].

### 2.3. LS

Li et al. [34] presented a LS method for segmentation in the presence of intensity inhomogeneities. Suppose that *Ω* is the image domain and *I* : *Ω* → *R* is a gray level image and also *Ω* = ∪_*i*=1_^*N*^ *Ω*_*i*_, *Ω*_*i*_∩*Ω*_*j*_ = *∅* → *i* ≠ *j*. The image can be modeled as

(11) $$\begin{array}{c} I=bJ+n, \end{array}$$

where *J* is the true image, *b* is the component that accounts for the intensity inhomogeneity, and *n* is additive noise. For the local intensity clustering, consider a circular neighborhood with a radius *ρ* centered at each point *y* ∈ *Ω*, defined by *O*_*y*_≜{*x* : |*x* − *y* | ≤*ρ*}. The partition {*Ω*_*i*_}_*i*=1_^*N*^ of *Ω* induces a partition of the neighborhood *O*_*y*_. Therefore, the intensities in the set *I*_*y*_^*i*^ = {*I*(*x*) : *x* ∈ *O*_*y*_∩*Ω*_*i*_} form the clusters, where *I*(*x*) is the image model. Now, we define a clustering criterion *ε*_*y*_ for classifying the intensities in *O*_*y*_. We need to jointly minimize *ε*_*y*_ for all *y* in *Ω* which is achievable by minimizing the integral of *ε*_*y*_ with respect to *y* over the image domain *Ω*. So, the energy formulation is as below:

(12) $$\begin{array}{c} \varepsilon =\int \varepsilon_{y}dy. \end{array}$$

It is difficult to solve the expression *ε* to minimize the energy. Therefore, we express the energy as LS function. LS function is a function that takes positive and negative signs, which can be used to represent a partition of the domain *Ω*. Suppose *ϕ* : *Ω* → *R* is a Level Set function. For example, for two disjoint regions,

(13) Ω1=x:ϕx>0,Ω2=x:ϕx<0.

For the case of *N* > 2, two or more LS functions can be used to represent *N* regions *Ω*_1_,…, *Ω*_*N*_. Now, we formulate the expression *ε* as a Level Set function:

(14) ε=∫∑i=1N∫ky−xIx−byci2dy·Miϕxdx,

where *M*_*i*_(*ϕ*) is a membership function. For example, for *N* = 2, *M*_1_(*ϕ*) = *H*(*ϕ*), *M*_2_(*ϕ*) = 1 − *H*(*ϕ*), and *H* is the Heaviside function, *c*_*i*_ is a constant in each subregion, and *k* is the kernel function defined as a Gaussian function with standard deviation *σ*. This energy is used as the data term in the energy of the proposed variational LS formulation which is defined by

(15) $$\begin{array}{c} F\left(\;\phi ,c,b\;\right)=\varepsilon \left(\;\phi ,c,b\;\right)+\nu L\left(\;\phi \;\right)+\mu R_{p}\left(\;\phi \;\right), \end{array}$$

where *ℒ*(*ϕ*) and *ℛ*_*p*_(*ϕ*) are the regularization terms. By minimizing this energy, the segmentation results will be obtained. This is achieved by an iterative process. As a result, the LS function encompasses the desired region.

This algorithm has also been used in the processing of fundus images [35, 36].

## 3. Segmentation of Intracorneal Layers

**Figure 2:**
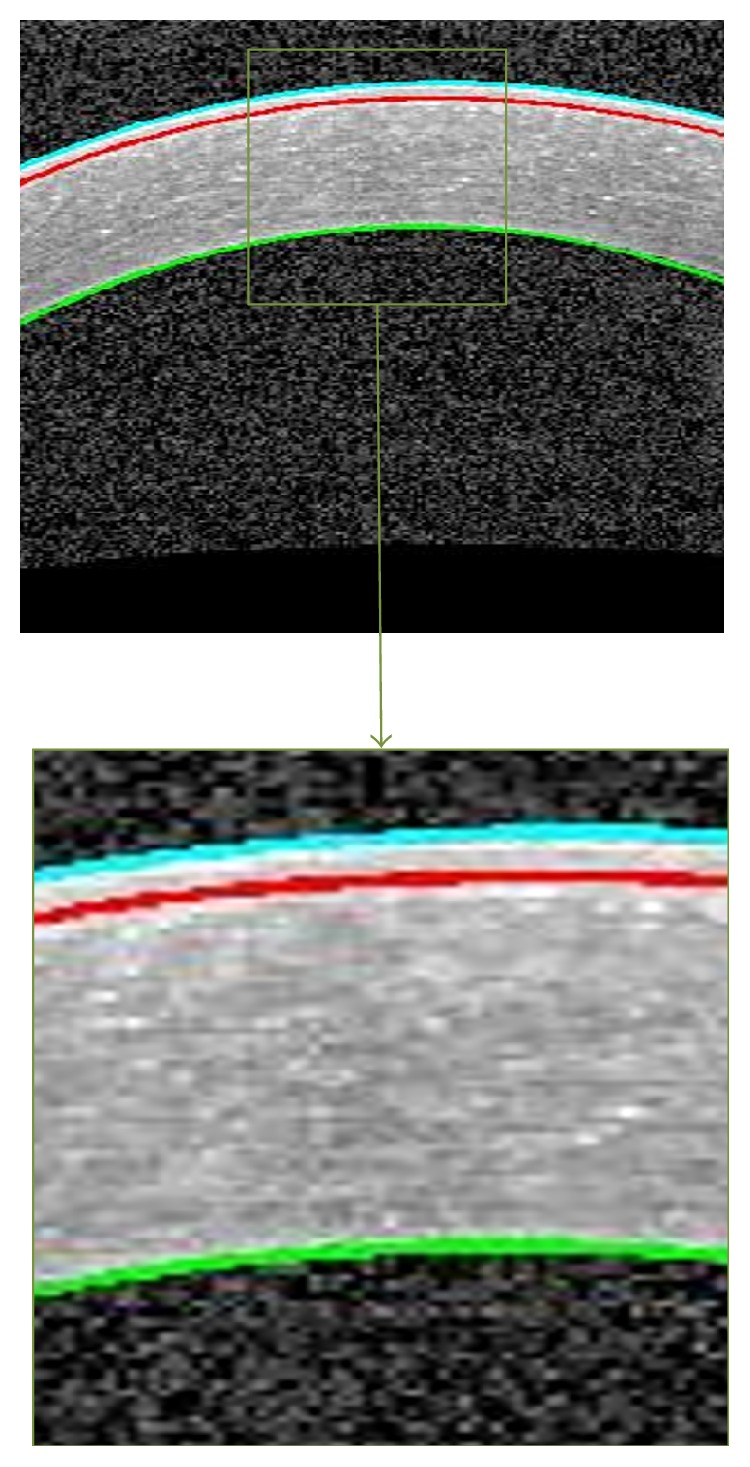
An example of segmented corneal image. The Epithelium boundary (cyan), the Bowman boundary (red), and the Endothelium boundary (green).

The important corneal layers, that is, Epithelium, Bowman, and Endothelium, are shown in Figure 2.

For production of thickness maps, after preprocessing, the implementation method for segmenting the desired boundaries using GMM, GC, and LS is investigated and the best segmentation method is chosen. Finally, using the segmentation results of all B-scans the intracorneal thickness maps are produced.

### 3.1. Preprocessing

Duo to the noise of the images and their low contrast, a preprocessing stage is proposed. Then, the mentioned algorithms are applied for segmenting the boundaries of Epithelium, Bowman, and Endothelium layers.

#### 3.1.1. Noise Reduction

**Figure 3:**
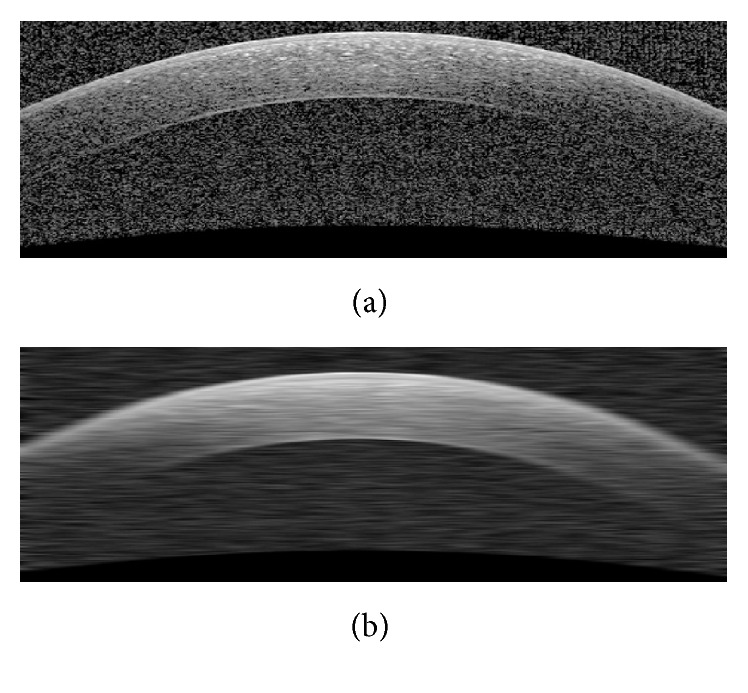
(a) Original image. (b) Denoised image using a Gaussian kernel [1 × 30] (std of 10).

The presence of noise in the OCT images causes errors in the final segmentation. To overcome this problem, we apply a low-pass filter via a Gaussian kernel to minimize the effect of noise. The kernel size of this filter which is going to be applied on OCT images and used as GMM inputs is [1 × 30] with std of 10. These kernel sizes in LS and GC are [1 × 20] (std of 20/3) and [1 × 5] (std of 5/3), respectively. The selected kernel sizes lead to uniformity of the image noise and prevent oversegmentation. These values for the kernel sizes and stds are obtained based on trial and error to have best results.  Figure 3 shows an example of denoising step.

#### 3.1.2. Contrast Enhancement

**Figure 4:**
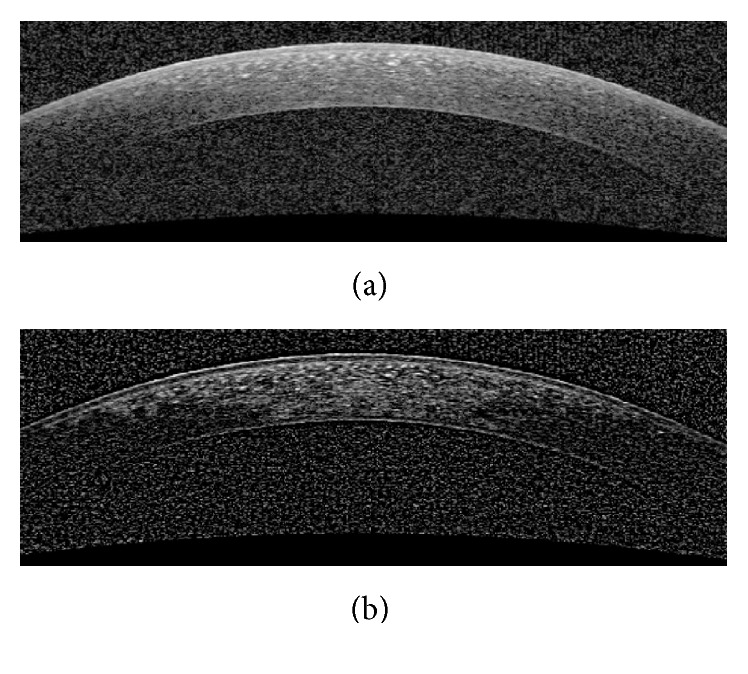
Contrast enhancement of original image for segmentation of Bowman boundary. (a) Original image. (b) The enhanced image.

OCT images usually have low contrast and we will be faced with a problem of segmenting the boundaries (especially the Bowman boundary). To enhance the contrast, we modified a method proposed by Esmaeili et al. [37] where each pixel *f*(*i*, *j*) of image is modified as follows:

(16) gi,j=2×fi,j−2×fmeanw+100×fmini,jfmaxi,j+1,

where *f*_mean_, *f*_min_(*i*, *j*), and *f*_max_(*i*, *j*) are, respectively, the mean, minimum, and maximum intensity values of the image within the square 10 × 10 window around each pixel (*i*, *j*).  Figure 4 shows an example of this process. As we can see, using this method nonuniform background is corrected on top of contrast enhancement.

#### 3.1.3. Central Artifact

**Figure 5:**
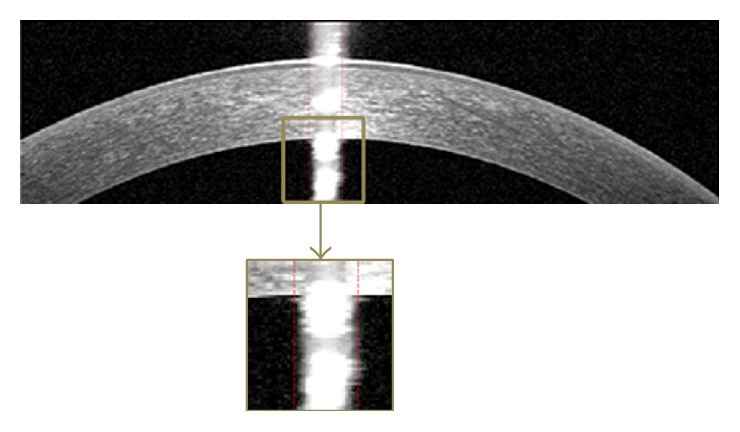
An example of central artifact positioning.

One of the most important artifacts in corneal OCT images is the central artifact that overcast corneal boundaries. So, reducing its effect causes the algorithm to be more successful. This artifact is nonuniform and we implement an algorithm that is robust to variations in width, intensity, and location of the central artifact. At first, to find abrupt changes in the average intensity, we accentuate the central artifact by median filtering the image with a [40 × 2] kernel. Then, we break the image into three equal width regions and suppose that the second region contains the central artifact. In the next step, we plot the average intensity of columns of the middle region. After that we look for the column where the difference intensity between this column and its previous column is more than 6.  Figure 5 shows an example of central artifact positioning.

### 3.2. Segmentation of Three Corneal Layer Boundaries with GMM

#### 3.2.1. Segmenting the Boundaries of Epithelium and Endothelium Layers

**Figure 6:**
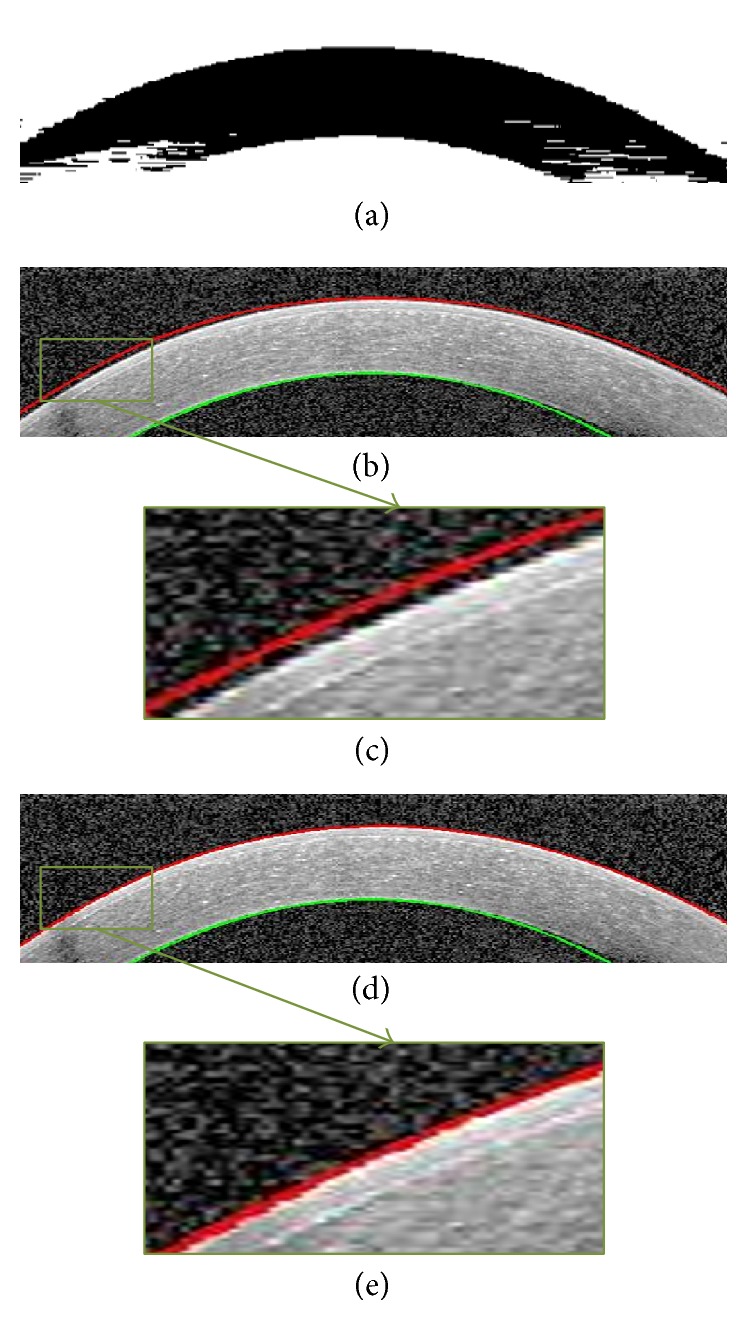
Segmentation of Epithelium and Endothelium boundaries. (a) GMM output. This is the image used for segmentation of desirable boundaries. (b) Segmentation result before correction. (c) Zoomed results of extracted Epithelium before correction. (d) Epithelium boundary after correction. (e) Zoomed results of extracted Epithelium after correction.

To obtain these two boundaries, we first reduce the image noise using the method introduced in Section 3.1.1. Then we give the denoised image to the GMM algorithm. Actually we use univariate distribution for image intensities and 2 components where one of them models the background and the next one models the main information between Epithelium and Endothelium layers.  Figure 6 shows the final result of GMM-based segmentation. We can see the approximate location of Epithelium and Endothelium boundaries which is obtained by choosing maximum responsibility factor in (4) and curve fitting to the boundaries. As it can be seen in Figure 6(c), the detected boundary is not exactly fitted to the border of Epithelium. So, the horizontal gradient of original image is calculated with help of the current boundary, choosing the lowest gradient in a small neighborhood (Figure 6).

#### 3.2.2. Correction of Low SNR Regions

**Figure 7:**
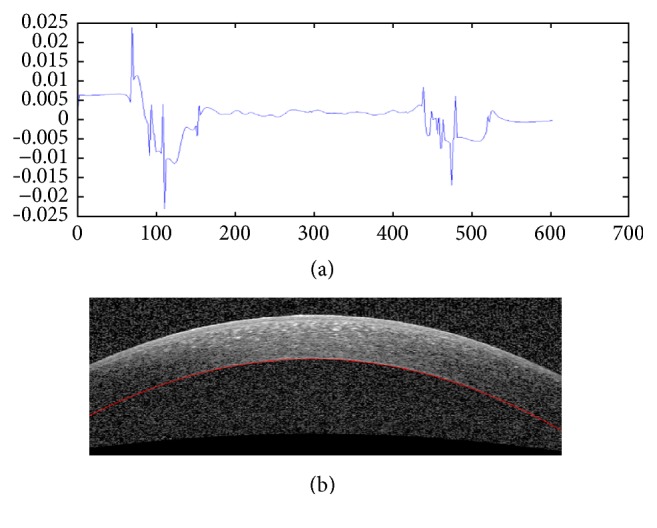
Extrapolation to low SNR regions. (a) The second derivative plot of Endothelium layer boundary to detect low SNR regions of this boundary. (b) Extrapolation result.

As we can see in Figure 6, the obtained Endothelium boundary in peripheral regions is not accurate because the SNR is low in these areas. So, extrapolation of central curve in these low SNR regions is proposed. By taking the second derivative of Endothelium boundary and searching around the middle of this vector the values below zero are found. It is observed that for low SNR regions there is a positive inflection in the second derivative. After finding the location of this positive inflection, by finding the coefficients of best polynomial of degree 4 fitted to correct estimated border a parabolic curve is obtained to estimate the Endothelium boundary in low SNR areas. To have a smooth enough curve for the final Endothelium boundary, a local regression using weighted linear least squares that assigns lower weight to outliers is used.  Figure 7 shows an example of extrapolation of low SNR regions using this method.

#### 3.2.3. Bowman Layer Segmentation

**Figure 8:**
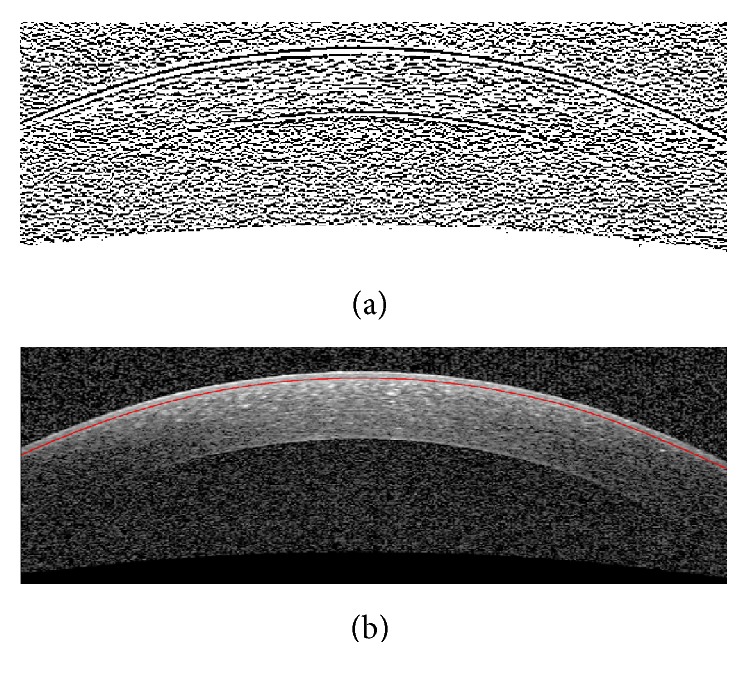
Segmentation of Bowman layer boundary. (a) Horizontal gradient of the enhanced image. (b) Final Bowman layer segmentation result.

Since in most cases the Bowman boundary is very weak we first enhance this boundary employing the contrast enhancement method explained in Section 3.1.2. After enhancement, the horizontal edges are obtained by applying Sobel gradient.  Figure 8 shows this procedure which can extract the Bowman boundary. Since we have obtained the Epithelium boundary (as described in Section 3.1), the Bowman boundary can be obtained by tracing Epithelium toward down to get a white to black change in brightness. However, unlike the middle of the Bowman boundary, the periphery may be corrupted by noise. To overcome this problem, similar to the proposed extrapolation method for low SNR areas in previous subsection, the outliers are corrected.

#### 3.2.4. Interpolation into the Central Artifact Location

**Figure 9:**
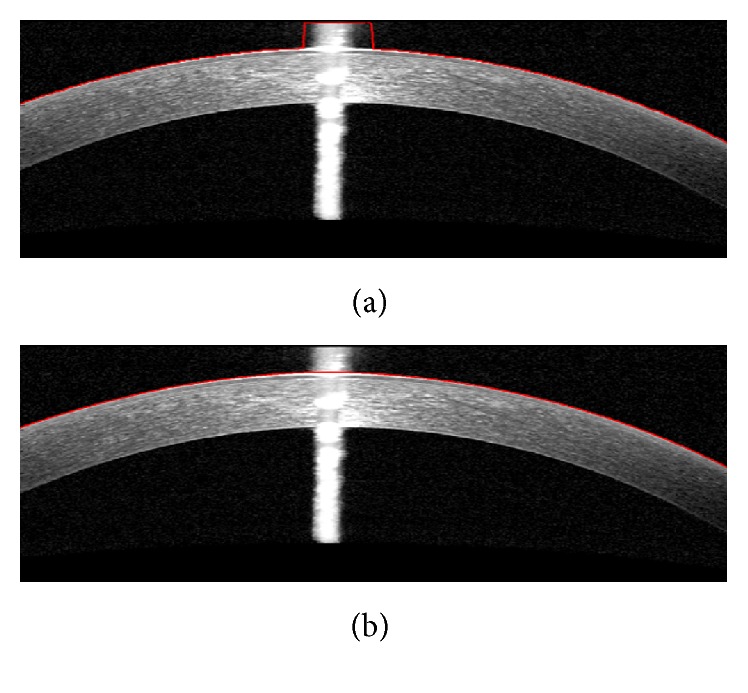
(a) Primary segmentation of Epithelium boundary that has failed in the central artifact. (b) The corrected Epithelium boundary.

Central artifact causes the estimated boundary to be imprecise. So, we first find the artifact location with the method elaborated in Section 3.1.3 and then we make a linear interpolation on the estimated boundary in this location, as shown in Figure 9.

### 3.3. Segmentation of Three Corneal Layer Boundaries with LS

#### 3.3.1. Segmenting the Boundaries of Epithelium and Endothelium Layers

**Figure 10:**
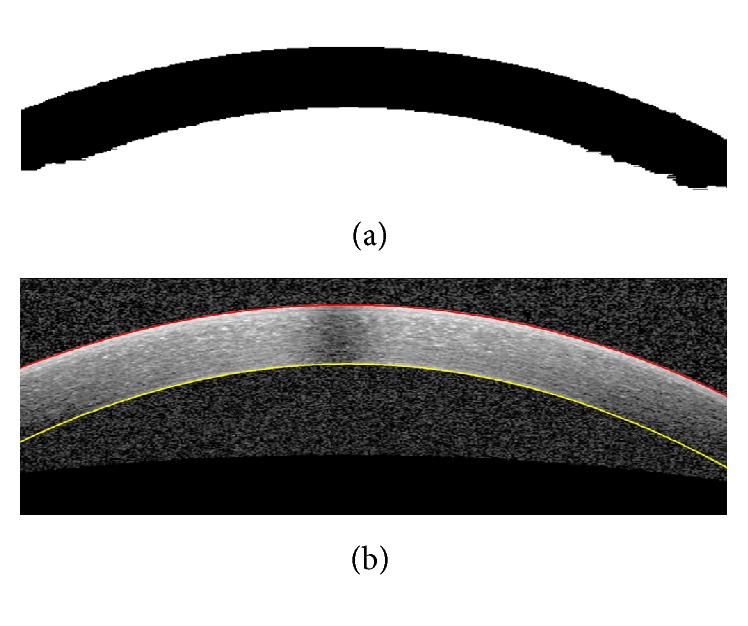
(a) Output of Level Set. (b) The estimated Epithelium and Endothelium boundaries.

To obtain these boundaries, we first denoise the images according to the method described in Section 3.1.1; then the LS algorithm is applied to the denoised image. The parameters are configured in this way: suppose *A* is equal to 255; then, *ν* = 0.01 × *A* ^2^, *σ* = 5, and *μ* = 1. The final result of LS shows the approximate location of these two boundaries. Extrapolation and interpolation are performed according to what we explained for GMM (Figure 10).

#### 3.3.2. Segmenting the Boundary of Bowman Layer

**Figure 11:**
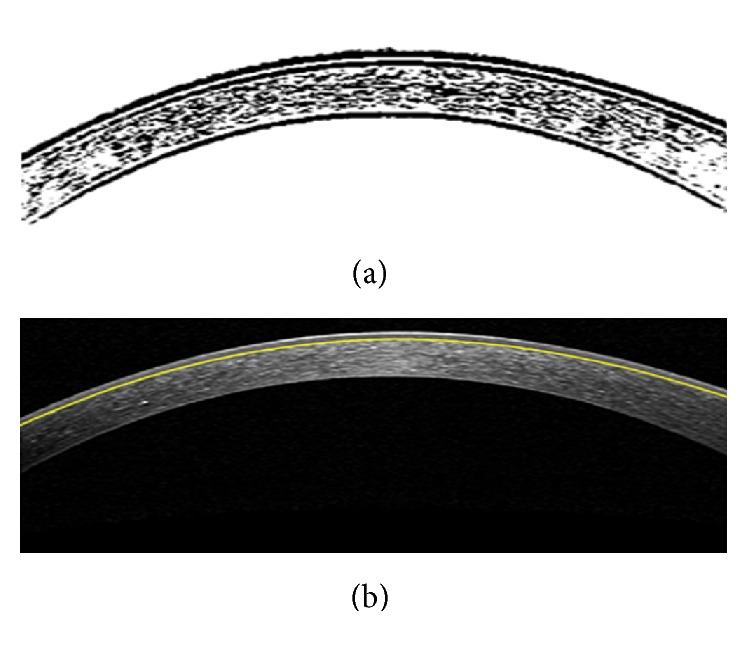
(a) Output of Level Set. (b) The estimated Bowman boundary.

For this purpose, to have an enhanced Bowman boundary the presented contrast enhancement method in Section 3.1.2 is performed. In the next step, we apply LS to this image with *ν* = 0.001 × *A* ^2^ and there is no change in other parameters. This value causes segmentation of much more details, in particular Bowman boundary.  Figure 11(a) is the output of LS and using this image we can localize the Bowman boundary similar to what we did in GMM. Bowman boundary can be finally detected with the help of Epithelium boundary (Figure 11(b)).

### 3.4. Segmentation of Three Corneal Layer Boundaries with GC

#### 3.4.1. Segmenting the Boundaries of Epithelium and Endothelium Layers

**Figure 12:**
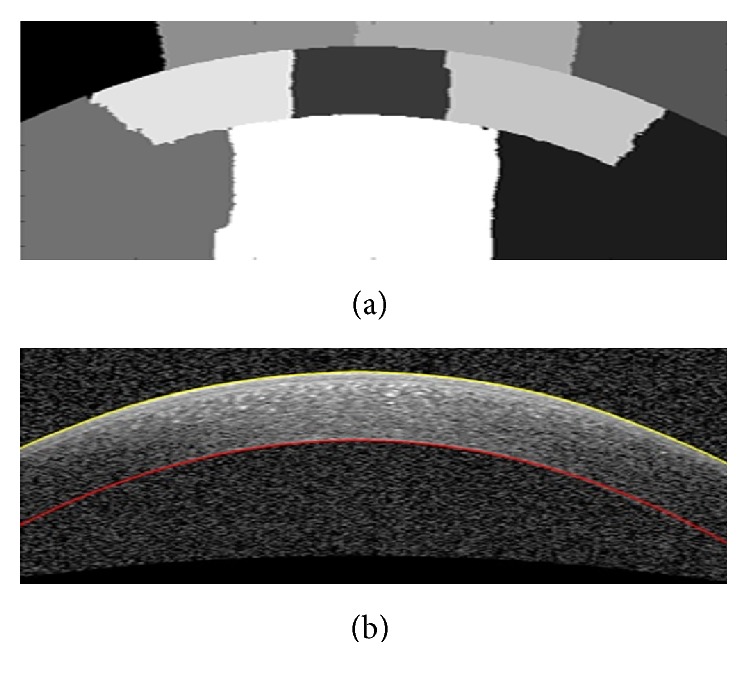
(a) Output of Graph Cut. (b) The estimated boundaries.

To obtain these boundaries, we first denoise the images according to the proposed method in Section 3.1.1. Then we apply GC considering the cutting area of 10.  Figure 12(a) shows the output image and we can achieve the desired boundaries by looking for a change in intensity starting from the first row and the last one. The extrapolation to low SNR regions and interpolation for the central artifact are like what was performed for GMM.

#### 3.4.2. Segmenting the Boundary of Bowman Layer

**Figure 13:**
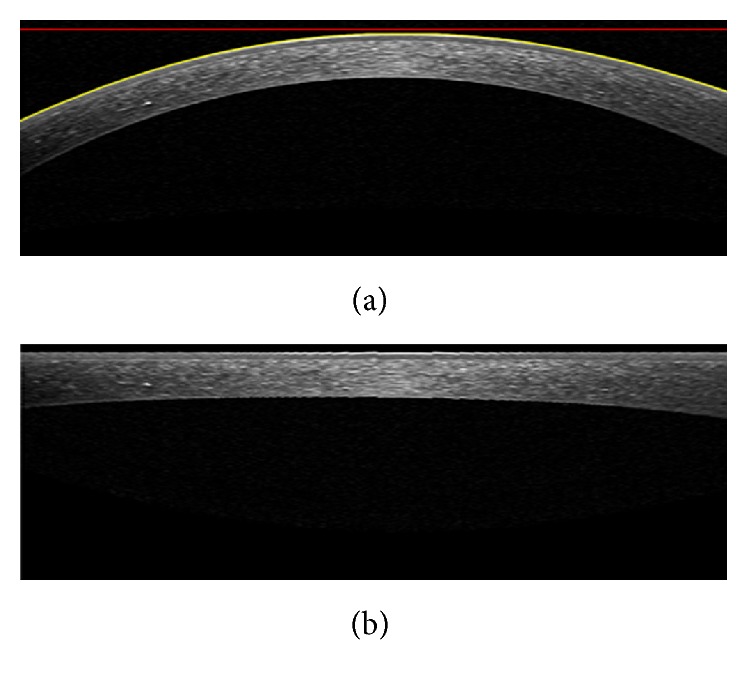
(a) The method to flattening the image using a horizontal line and Epithelium boundary. (b) Flat image.

**Figure 14:**
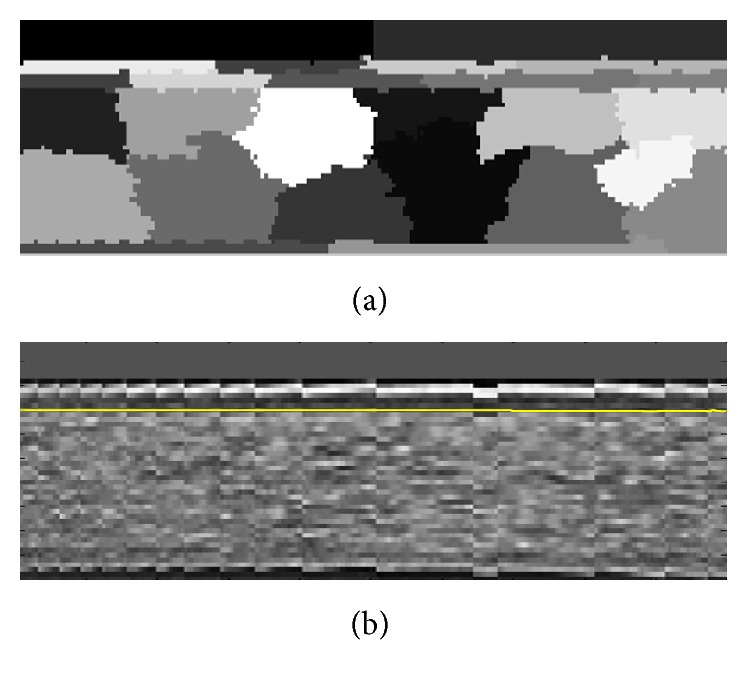
(a) Output of Graph Cut for the restricted area to the Bowman boundary. (b) Bowman boundary estimation in the middle based on the Epithelium boundary.

**Figure 15:**
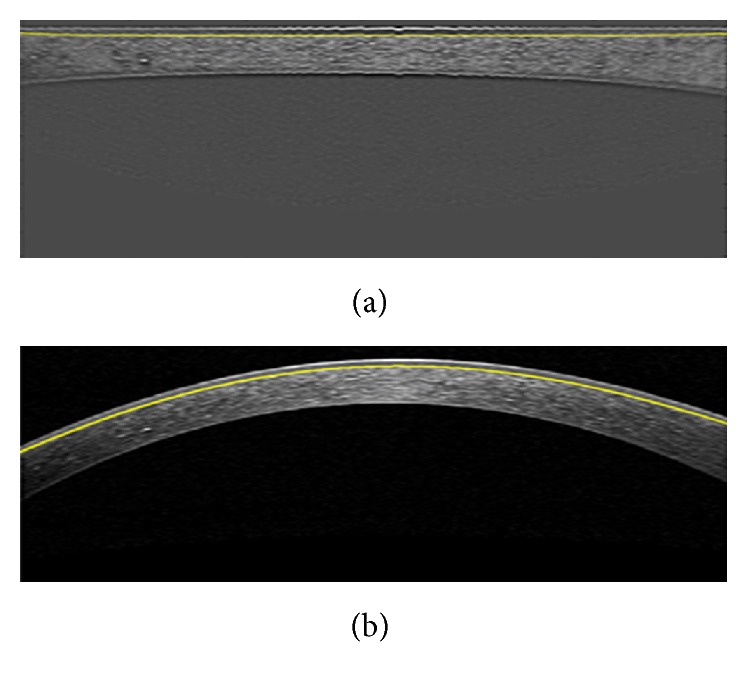
(a) Linear extrapolation of estimated Bowman boundary in the middle of the image. (b) Final result of Bowman boundary segmentation.

For this purpose, similar to other two algorithms, we first enhance the original image. In the next step, we flatten this image based on the obtained Epithelium boundary from the previous step and with respect to a horizontal line as shown in Figure 13(a). The flattening algorithm works by shifting the pixel positions which are under the Epithelium boundary to be fitted on a horizontal line (Figure 13(b)). The Bowman boundary can be tracked better in horizontal setup. Applying the algorithm to the entire flattened image is both time consuming and less reliable. So, the image is divided into three parts, and the algorithm is applied to the restricted area to the Bowman boundary and based on the Epithelium boundary, the desired boundary is achieved (Figure 14). The estimated boundary in the middle area is accurate and the linear extrapolation of this boundary to the image width is then performed as shown in Figure 15 (the estimated boundaries in the two other parts usually are not accurate).

## 4. Three-Dimensional Thickness Maps of Corneal Layers

In the previous section, 3 methods for intracorneal layer segmentation were explained. In this section these methods are compared and the best one is picked to produce the intracorneal thickness maps by employing the segmentation results of all B-scans.

### 4.1. Comparing Segmentation Results of GMM, LS, and GC Methods

In this study, we used corneal OCT images taken from 15 normal subjects. Each 3D OCT includes 40 B-scans of the whole cornea. The images were taken from the Heidelberg OCT-Spectralis HRA imaging system in NOOR ophthalmology center, Tehran. To evaluate the robustness and accuracy of the proposed algorithms, we use manual segmentation by two corneal specialists. For this purpose, 20 images were selected randomly from all subjects. We calculated the unsigned and signed error of each algorithm against manual results.

The boundaries were segmented automatically using a MATLAB (R2011a) implementation of our algorithm. A computer with Microsoft Windows 7 x32 edition, intel core i5 CPU at 2.5 GHZ, 6 GB RAM, was used for the processing. The average computation times for GMM, LS, and GC were 7.99, 19.38, and 17.022 seconds per image, respectively. The mean and std of unsigned and signed error of the mentioned algorithms were calculated and are shown in Tables 1 [Table tab2] [Table tab3] [Table tab4] [Table tab5]–6. The mean and std of unsigned error are defined as follows:

(17) merror=mean∑i=1nbmanuali−bautoi,σerror=standard  deviation∑i=1nbmanuali−bautoi,

where *m*_error_ and *σ*_error_ are the mean and std of unsigned error between manual and automatic methods, *n* is the number of points to calculate the layer error (width of the image), and *b*_manual_ and *b*_auto_ are the boundary layers which are obtained manually and automatically, respectively. The mean value of manual segmentation by two independent corneal specialists is considered as *b*_manual_ in the above equations and the interobserver errors are also provided in Tables 1 [Table tab2] [Table tab3] [Table tab4] [Table tab5]–6. The direct comparison of these values with the reported errors shows that the performance of GMM algorithm is more acceptable in comparison with manual segmentation.

With study of the presented results such as the lower segmentation error for each of the layer boundaries and time spent for segmenting the borders, it is observed that GMM method compared to the position differences between two expert manual graders (Table 7) give better results compared with two other methods.

### 4.2. Producing Intracorneal Thickness Maps

**Figure 16:**
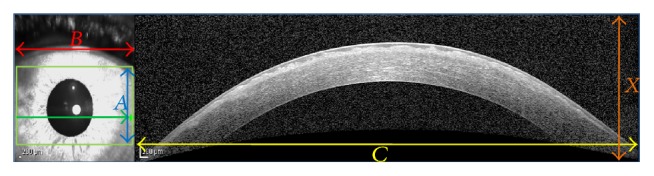
The method to calculate the number of pixels for interpolation.

**Figure 17:**
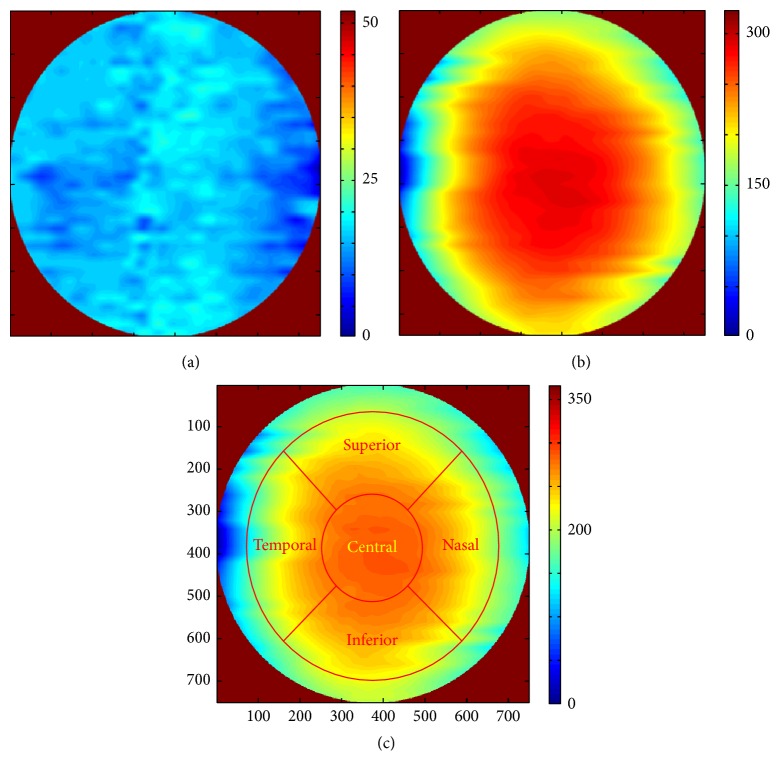
3D thickness maps of a normal subject. (a) Whole cornea; (b) layer 1; (c) the layer created by Bowman and Endothelium boundaries (the zones are defined in concentric circles with diameters equal to 2 and 5 millimeters, as central, superior, inferior, nasal, and temporal zones).

According to what is described above, GMM was selected as the optimal method for segmentation of corneal layers. Therefore, using GMM method, the corneal layer boundaries in the volumetric images of each subject are segmented. The segmented values are then interpolated (as described below) to create the thickness maps. To illustrate the layer thickness, we first subtract the boundaries which create a layer and an interpolation is performed between two consecutive volumetric images. The number of pixels between two successive images for interpolation is calculated as shown in Figure 16. Considering pixel values of *C* and *X*, the equation between *C* and *B* and scale signs in right and left images, the value pixels which correspond to of *A* can be found. This value ($\overset{´}{A}$) should be used in interpolation and the number of interpolation pixels is equal to $\overset{´}{A}/number\;\;of\;\;slices$. It can be shown that the OCT slices cover an area of 6 millimeters in width and height.  Figure 17 shows the 3D thickness maps of corneal layers of a normal subject.

Mean and standard deviation of the thickness values for normal subjects in epithelial, stromal, and whole cornea are calculated in central, superior, inferior, nasal, and temporal zones (cantered on the centre of pupil). The zones are defined in concentric circles with diameters equal to 2 and 5 millimeters (Figure 17(c)). The thickness averages (±population SD) are shown in Table 8.

## 5. Conclusion

In this paper we compared three different segmentation methods for segmentation of three important corneal layers of normal eyes using OCT devices. According to good performance of the GMM method in this application, we chose GMM as the optimal method to calculate the boundaries. The proposed method is able to eliminate the artifacts and is capable of automatic segmentation of three corneal boundaries.

In the next step, we obtained the thickness maps of corneal layers by interpolating the layer information. Mean and standard deviation of the thickness values in epithelial, stromal, and whole cornea are calculated in 3 central, superior, and inferior zones (cantered on the centre of pupil). To the best of our knowledge, this is the first work to find the thickness maps from a set of parallel B-scans and all of the previous methods in construction of thickness map from OCT data use pachymetry scan pattern [13, 14, 16].

## Figures and Tables

**Table 1:** Mean and standard deviation of unsigned error in corneal layer boundary segmentation between automatic and manual segmentation using GMM.

Corneal layer boundary	Mean difference (*µ*m)	Standard deviation (*µ*m)
Epithelium boundary	2.99464	2.0746
Bowman boundary	3.79742	2.89542
Endothelium boundary	7.1709	6.74696

**Table 2 tab2:** Mean and standard deviation of signed error in corneal layer boundary segmentation between automatic and manual segmentation using GMM.

Corneal layer boundary	Mean difference (*µ*m)	Standard deviation (*µ*m)
Epithelium boundary	0.09922	3.36446
Bowman boundary	1.59654	3.81546
Endothelium boundary	0.39688	8.05486

**Table 3 tab3:** Mean and standard deviation of unsigned error in corneal layer boundary segmentation between automatic and manual segmentation using level set.

Corneal layer boundary	Mean difference (*µ*m)	Standard deviation (*µ*m)
Epithelium boundary	4.40176	2.5256
Bowman boundary	7.00854	3.3825
Endothelium boundary	6.97246	6.765

**Table 4 tab4:** Mean and standard deviation of signed error in corneal layer boundary segmentation between automatic and manual segmentation using level set.

Corneal layer boundary	Mean difference (*µ*m)	Standard deviation (*µ*m)
Epithelium boundary	−3.22014	3.25622
Bowman boundary	−5.6375	4.70844
Endothelium boundary	−0.14432	8.69528

**Table 5 tab5:** Mean and standard deviation of unsigned error in corneal layer boundary segmentation between automatic and manual segmentation using graph cut.

Corneal layer boundary	Mean difference (*µ*m)	Standard deviation (*µ*m)
Epithelium boundary	4.08606	2.4354
Bowman boundary	6.23282	4.20332
Endothelium boundary	7.9376	7.28816

**Table 6:** Mean and standard deviation of signed error in corneal layer boundary segmentation between automatic and manual segmentation using graph cut.

Corneal layer boundary	Mean difference (*µ*m)	Standard deviation (*µ*m)
Epithelium boundary	−2.76012	3.34642
Bowman boundary	−0.32472	6.55754
Endothelium boundary	7.18894	7.9376

**Table 7:** The position differences between the two expert manual graders.

Corneal layer boundary	Mean difference (*µ*m)	Standard deviation (*µ*m)
Epithelium boundary	2.9766	2.73306
Bowman boundary	3.58996	2.71502
Endothelium boundary	4.81668	4.88884

**Table 8:** 

Corneal layers	Central	Superior	Inferior	Nasal	Temporal
Epithelial layer					
Averages	42.9352	43.6568	42.9352	41.943	43.9274
SD	4.2394	2.9766	9.3808	6.5846	3.157
Stromal layer					
Averages	463.2672	481.9386	507.2848	488.3428	497.904
SD	33.7348	38.335	38.9664	32.1112	37.6134
Whole cornea					
Averages	489.9664	529.2034	535.0664	529.8348	534.6154
SD	39.3272	43.9274	38.1546	34.8172	37.5232
